# Characteristics of communication guidelines that facilitate or impede guideline use: a focus group study

**DOI:** 10.1186/1471-2296-8-31

**Published:** 2007-05-16

**Authors:** Wemke Veldhuijzen, Paul M Ram, Trudy van der Weijden, Susan Niemantsverdriet, Cees PM van der Vleuten

**Affiliations:** 1 Department of General Practice, Maastricht University, Debeyeplein, Maastricht, The Netherlands; 2 Vocational Training Centre for General Practice, Debeyeplein, Maastricht, The Netherlands; 3 Centre for Quality of Care Research, Maastricht University, Debeyeplein, Maastricht, The Netherlands; 4 Department of Educational Development and Research, Maastricht University, Universiteitssingel, Maastricht, The Netherlands

## Abstract

**Background:**

The quality of doctor-patient communication has a major impact on the quality of medical care. Communication guidelines define best practices for doctor patient communication and are therefore an important tool for improving communication. However, adherence to communication guidelines remains low, despite doctors participating in intensive communication skill training. Implementation research shows that adherence is higher for guidelines in general that are user centred and feasible, which implies that they are consistent with users' opinions, tap into users' existing skills and fit into existing routines. Developers of communication guidelines seem to have been somewhat negligent with regard to user preferences and guideline feasibility. In order to promote the development of user centred and practicable communication guidelines, we elicited user preferences and identified which guideline characteristics facilitate or impede guideline use.

**Methods:**

Seven focus group interviews were conducted with experienced GPs, communication trainers (GPs and behavioural scientists) and communication learners (GP trainees and medical students) and three focus group interviews with groups of GP trainees only. All interviews were transcribed and analysed qualitatively.

**Results:**

The participants identified more impeding guideline characteristics than facilitating ones. The most important impeding characteristic was that guidelines do not easily fit into GPs' day-to-day practice. This is due to rigidity and inefficiency of communication guidelines and erroneous assumptions underpinning guideline development. The most important facilitating characteristic was guideline structure. Guidelines that were structured in distinct phases helped users to remain in control of consultations, which was especially useful in complicated consultations.

**Conclusion:**

Although communication guidelines are generally considered useful, especially for structuring consultations, their usefulness is impaired by lack of flexibility and applicability to practice routines. User centred and feasible guidelines should combine the advantages of helping doctors to structure consultations with flexibility to tailor communication strategies to specific contexts and situations.

## Background

Most doctors are familiar with clinical guidelines for medical technical purposes, such as managing diabetes or hypertension. Guidelines for doctor patient communication are less commonly used, however, although there appears to be no reason why this domain should be excluded from guideline development. The doctor patient relationship is an important factor in health care and the quality of doctor-patient communication has been shown to impact strongly on the quality of medical care. Good doctor patient communication enhances patient satisfaction and work satisfaction of doctors, moreover it lowers use of health care resources, and improves health outcomes of patients [1-14]. Many authors have presented models, frameworks, guides or guidelines for best practices in doctor patient communication which can be used to shape the content of communication training courses [6,15-20].

Henceforward we will use the term 'communication guidelines' to refer to all documents containing evidence based recommendations for doctor patient communication, even when another designation is used in the document itself or in the literature. We use the term guideline because it has been clearly defined and because of the existing knowledge about implementing guidelines.

Clinical guidelines help practitioners to practise evidence based medicine in routine practice and are considered to be important tools for improving the quality of health care [21-23]. However, doctors' adherence to guidelines generally varies and can be quite low sometimes [24-27]. In the case ofdoctor patient communication, doctors have shown low adherence to guidelines, despite extensive training [9,13,28-32].

Guideline implementation has been addressed by many recent studies. User centredness and feasibility of guidelines have been found to facilitate implementation [23,25,33]. This implies that it is important for guidelines to be in agreement with users' opinions, tap into users' existing skills and fit into existing routines. These characteristics can be enhanced by involving future guideline users in guideline development and evaluation [23,25].

Implementation research has primarily focussed on medical technical guidelines. Doctor patient communication has been relatively neglected, with a noticeable dearth of studies on the applicability of communication guidelines [34].

Efforts to address implementation problems of communication guidelines have focused primarily on improving the implementation strategy by optimising training methods. Optimal training methods combine explanation of communication theory with many opportunities for students to practise communication skills, within a longitudinal training programme [35-40].

Criticisms of the applicability of communication guidelines have been countered by suggestions for changing practitioners' attitudes or even the organisation of health care, but have rarely resulted in revisions of communication guidelines [41-44]. There is little evidence that doctors' views and preferences are being sought and taken into account when communication guidelines are being developed and evaluated [16,45]. Broadly speaking, there are indications that the use of communication guidelines can be promoted by making them more user centred and feasible.

We conducted ten focus groups interviews in which guideline users discussed characteristics of communication guidelines that facilitated or impeded their use. We explored ways to develop more user centred and applicable guidelines.

## Methods

### Participants

Since communication skills training has a long history in general practice, we conducted our study within the setting of the Dutch centres for postgraduate specialist training in general practice. These centres are located in the Departments of Family Medicine of the eight Dutch Faculties of Medicine. We sought the opinions of different groups of guideline users, because we hoped that this would yield a rich diversity of experiences. We asked the eight GP training centres to invite experienced GPs, communication trainers (GPs and behavioural scientists) and communication learners (GP trainees and medical students) to take part in a focus group interview at the training centre. Because not all centres use the same guidelines, the focus group participants differed as to which guidelines they used and for which purpose (clinical practice, learning, teaching) they used it.

To elicit unbiased opinions from participants with lower status (i.e. trainees and students), including opinions that might be disagreeable to higher status participants (i.e. teachers), we organised three trainee-only focus groups in addition to the mixed group sessions planned for each of the eight training centres. The three trainee-only groups consisted of a group of first year trainees, a group of last year trainees and a combined group of first and last year trainees. The participants in the third group were selected based on their teachers' expectations that they would voice strong opinions, both positive and negative, about communication training.

### Data collection

The moderator of the focus groups was experienced in qualitative interviews (WV). Each group focused on the guideline for GP patient communication that was most commonly used in the training centre where the interview was held (see appendix 1). We adhered to the definition of a communication guideline as a document 'containing recommendations, guidance and instructions about doctor patient communication, intended to support daily practice in health care and based on results of scientific research and the consequent discussion and formation of opinion, aimed at the explicit statement of good medical practice' [46]. Participants were invited to discuss guideline features that facilitated or impeded guideline use as well as ways of improving the communication guidelines.

### Data analysis

All focus group discussions were audiotaped and transcribed. The transcriptions were analysed by WV using specialised software (Atlas-ti). All phrases relating to the research questions and interview topics were coded and the resulting codes were placed in code networks displaying the relationships between the codes, to facilitate the formation of concepts, categories and hypotheses. Guideline characteristics that impeded and guideline characteristics that facilitated guideline use as well as suggestions for improvement were analysed for common themes and categorised according to these themes. To maximise richness of interpretation six of the ten focus group interviews were analysed independently by three different researchers with different backgrounds(PR, TvdW, and SN). These analyses were compared with those of the first researcher (WV) and differences of interpretation were discussed until consensus was reached.

## Results

### Participation

Because one GP training centre was unable to participate due to time constraints, we organised one focus group interview in each of seven training centres. Additionally, three trainees-only sessions were organised. In all, seven mixed focus group sessions and three trainee-only sessions were held. The attendants of the mixed groups were: six experienced GPs, nineteen communication trainers (eight experienced GPs, eleven behavioural scientists), seven GP trainees and four medical students. Twenty-seven GP trainees attended the trainee only groups. Mean duration of the focus group sessions was 90 minutes. All sessions were characterised by animated discussions.

### Communication guidelines

Four training centres use the same communication guideline and three centres use a different guideline each (see appendix 1). This meant that we obtained results for four different guidelines. All guidelines pertain to general consultations and provide recommendations for consecutive phases of the consultation. More detailed descriptions can be found in Appendix 2.

Which guideline was discussed did not seem to have a major impact on the discussion. There were no pronounced differences between the guidelines with regard to barriers and facilitating factors that emerged during the group discussions. The behavioural science teachers tended to hold the most positive opinions of the guidelines and GP trainees were the most critical judges. There was no category of opinion, however, that was mentioned by one group only, or that was not mentioned by one of the groups, besides the differences in guideline use between learners and GPs, described in the next section.

### Guideline use

While discussing impeding and facilitating characteristics of the guidelines, the guideline users mentioned different ways of using a guideline. Because this offers valuable insight into users' needs and how these affect impeding and facilitating characteristics, we will first focus on guideline use.

The participants described six different uses of the communication guideline (Table 1). Students and some trainees were the only ones to report full guideline use. Experienced GPs and GP trainers generally used only parts of the guideline, i.e. only those recommendations they thought were appropriate for a particular situation.

**Table 1:** Guideline use

**Integral or partial use**	**Type of use**	**Description**
Partial use	Selective use	The guideline is used when it is considered to be especially useful, e.g. when the consultation is not going well.
Partial use	Fragmented use	Only some of the recommendations in the guideline are used. Which recommendations are used depends on: doctor characteristics (personal style, experience, goals), perceived patient characteristics (clarity, assertiveness), type of consultation (first consultation, follow-up visit), and type of complaint (somatic, psychosocial).
Partial use	Modified use	Most of the recommendations in the guideline are used, with some deliberate additions and/or omissions.
Integral use	Dispersed use	All recommendations in the guideline are used, dispersed over several consultations
Integral use	Implicit use	All recommendations in the guideline are used, but less explicitly and with more non-verbal communication than is recommended.
Integral use	Full use	All the recommendations in the guideline are used in a single consultation, mostly in the recommended order.

Types of guideline use described by the participants in the focus groups

It's just my toolkit. For a nail you use a hammer and when a screw turns up you start looking for a screwdriver.

GP trainee/UVA

It's some sort of theoretical schema for a consultation from which you use what you need at the time. What you don't need you simply leave out.

GP trainer/VU

It's a framework in your mind which you use to structure the interview and it depends on the person sitting opposite you and their wishes and needs how you proceed.

Behavioural scientist/UVA

The desirability of using only parts of a guideline was debated. Some argued that using only those recommendations that help doctors achieve their objective in a particular consultation is the most professional usage of communication guidelines. Other participants doubted whether doctors are capable of making the right choices when using only parts of a guideline. They cautioned against inaccurate assumptions and incomplete information.

The fact that a GP conducts a consultation in a purposeful manner. That he can change his goal because the patient suddenly changes his appeal for help. Noticing that and acting upon it, changing your goal and then working towards it. All that is part of professionalism.

Behavioural scientist/Rotterdam

### Facilitating and impeding guideline characteristics

The greater part of the discussions was devoted to guideline characteristics that impeded guideline use. When invited to discuss facilitating characteristics, participants frequently digressed and turned the discussion to impeding characteristics instead. Two central themes emerged from these discussions. The first one was 'Guideline development'. Within this theme, two sub-themes can be distinguished: 'Procedural development flaws' and 'Assumptive flaws'. The second central theme was 'Impact of guideline use' with the sub-themes: 'Impact on consultation process' and 'Other impact'. Table 2 categories the impeding and facilitating guideline characteristics in accordance with these themes. We will discuss them in the following section.

**Table 2:** Facilitators and barriers

**Theme**	**Guideline characteristics**
Procedural development flaws	- Supporting evidence is not convincing
	- Lack of instructions for use
	- Guideline developers are not representative of the target group
Assumptive flaws	*Erroneous assumptions about patients*
	- Not all patients are equal negotiating partners.
	- Not all patients have a background in Western culture.
	- Not all patients present with a new, well defined medical complaint.
	- Not all patients come by themselves.
	*Erroneous assumptions about doctors*
	- Not all communication is verbal.
	- Doctors need to be in charge.
	- Experienced doctors communicate differently.
	- Doctors want to have the opportunity to express a personal interest in their patients.
	- Using the guideline is too energy consuming
	*Erroneous assumptions about the situation*
	- Different situations need different approaches.
	- The guideline does not support long-term patient management strategies.
Impact on the consultation process	+ More grip on the consultation;
	+ More clarity for patients;
	- Less focus on the 'here and now'.
Other impact	+ Higher quality of consultations;
	+ Does justice to both patient and doctor;
	+ Less chance of jumping to conclusions;
	+ Fewer unreasonable patients;
	- Loss of time;
	- Loss of natural interaction and personal style;
	- Creating anxiety in patients.

Facilitating factors (+) and barriers (-) to guideline use, by theme

### Guideline development

#### Procedural development flaws

The users were of the opinion that procedural errors made during guideline development compromised the quality of the guidelines. Three types of development flaws were distinguished.

##### The supporting evidence is not convincing

The users thought that the recommendations were consensus based rather than evidence based. Moreover, the effectiveness of the guideline had not been tested in general practice and the underlying assumptions and beliefs were not described.

##### Lack of instructions for use

The users noted a lack of information as to how and in which situations the recommendations were to be used or which of the recommendations were expected to be used in any case, regardless of the situation.

You could even say that the models need good instructions about how to use the model. And we don't really have those. For instance, instructions telling you what's important, in such a way that you can recognise which phase of the model or the consultation you're at. And when you're struggling with a certain phase, those are the parts you can look at to get through it. And which parts you should always include and which parts are optional.

GP trainer/UM

##### Guideline developers are not representative of the target group

The users thought that social scientists were overrepresented among guideline developers and GPs underrepresented. The GPs felt that guideline developers had priorities that were different to theirs'.

#### Assumptive flaws

Users felt that the guidelines were somewhat artificial and reflected assumptions with little relevance to day-to-day practice. They felt that using the complete guideline was not helpful or that the guideline was of no use at all in many situations.

Well, what I wanted to say is that the problem here is that it's a very general schema and it is also just bristling with assumptions. And because of that funnily enough again it's not a general schema. And a lot of the problems you see in general practice they just don't fit into the schema.

GP trainer/VU

Participants said that some of the assumptions about patients, doctors and the situation in a consultation were incorrect.

##### Erroneous assumptions about patients

###### Not all patients are equal negotiating partners

The users mentioned that for patients to be equal negotiating partners, as assumed in the guideline, they should be intelligent, knowledgeable about health and health care organisation, mentally sane, have good self-knowledge, be assertive and act responsibly. Experience has taught doctors that many patients are unable to fully verbalise their requests for help, are claiming or unwilling to take responsibility for their own health. Patients are often surprised when their GP explores their request for help. Some patients do not appreciate it when the GP explores their emotions or beliefs, others do not want to participate in decision making.

I think that for a perfect exploration of the request for help you need a perfect patient, they just don't exist.

GP trainee/Nijmegen

Sometimes I hear people say, people without medical training: When I go and see my GP he asks me what I think about it. I think that 's just a lot of nonsense!

GP trainee/Nijmegen

###### Not all patients have backgrounds in Western culture

Patient responsibility is a central feature in Western perspectives on health care, whereas non-Westerners often expect a more authoritarian doctor and have higher expectations of the medical interventions they are being offered.

Language is not the problem, but those people have such a different attitude towards doctors. They want you to give a clear message, just tell me what has to be done.

GP trainee/UVA

###### Not all patients present with a new, well-defined medical complaint

Some complaints may be clear, but not readily identifiable as medical problems. Patients with chronic disease frequently do not have new complaints but only longstanding ones. Some patients have are not able to communicate clearly what their complaint actually is.

No, I find with somatic complaints it's easier to use than when a person has some sort of relationship problem for instance.

GP trainee/Nijmegen

###### Not all patients come by themselves

Patients are often accompanied by relatives or partners, who need to be attended to as well.

##### Erroneous assumptions about doctors

###### Not all doctor patient communication is verbal communication

Many things are checked or communicated non-verbally, for example by a questioning look or by remaining silent instead of a verbal exploration of the reason for the visit.

###### Doctors need to be in charge, sometimes

The guidelines are patient centred. Doctors sometimes feel that as a result they do not have sufficient opportunity to structure consultations when patients' stories are very long-winded and unstructured, support anxious or indecisive patients or set boundaries when patients behave inappropriately.

You also get very dominant people who refuse to be guided. So then you can keep returning to the MAAS-Global but they will always go back to their own story. And well, then it's really of no use whatsoever.

GP trainee/Nijmegen

Wouldn't you just like it now and again to say to a person who is being very rude and claiming: no, you may come back when you can behave properly. Without exploring what this person really wants.

GP trainee/VU

###### Experienced doctors communicate differently

Their long-standing relationships with patients help experienced doctors to understand their patients' requests for help.

Well, doctors do actually very much go on their first impressions, especially when you have had a patient in your practice for many years, so yes, it's only logical that you will pay more attention to those aspects in deciding what to do and what not to do.

GP trainer/VU

###### Doctors want to express a personal interest in their patients

The users said they wanted to be able to express their *personal interest *in their patients' lives and welfare, beyond the scope of the medical reason for the consultation.

###### Too energy consuming

It takes too much energy for a doctor to explore the emotional welfare of all his patients in depth.

Besides it's also true, I think that's actually even more of a problem, when I look at the 2650 people on my list and I'm truly interested in what's going on for all of them emotionally, then I'm putting myself in God's place, I think. That is impossible.

GP trainer/Nijmegen

##### Erroneous assumptions about the situation

###### Different situations need different approaches

Doctors modify their communication patterns according to their goals within a specific consultation, actual or perceived patient characteristics and type of complaint. This leads to communication strategies that are tailored to the consultation at hand. Doctors feel that most of the recommendations in guidelines are not suitable for every situation and that the sequence advocated by the recommendations is often not appropriate for a particular consultation. They feel that the guidelines do not afford them sufficient flexibility to achieve efficient and effective communication. This barrier is mentioned specifically when participants explain why they do not adhere to communication guidelines.

I think that the model can be very helpful for a classic or complicated consultation comprising a lot of different aspects, but whenever you're dealing with only a part of a problem you won't do that.

GP trainer/Nijmegen

###### The guideline does not support long-term patient management strategies

The guidelines offer recommendations for a single consultation, not for a series of consultations about a single problem. They do not support long-term follow-up strategies.

### Impact of guideline use

#### Impact on the consultation process

The consultation process is the most strongly affected by the structure that the guideline offers. Structure is the most appreciated guideline feature. The users say they appreciate the presentation of the consultation phases in chronological order and even more so the fact that the phases are conceptually different.

##### The phased structure enables doctors to keep a firm grip on a consultation

The phased structure gives doctors a better overview of the consultation. This enables them to improve structure and management of consultations, especially more complex ones.

Sometimes everything is going smoothly but there are also very difficult consultations and then I take a step back, then I see that model before me and then I know where I am in the consultation.

GP trainee/UVA

When you structure the interview like that then it becomes very easy to manage time, whereas when things are becoming just one big mess, it's really hard to finish within 10 minutes.

GP trainer/VU

##### More clarity for patients

Both the structure and the recommended summarisations help to create more clarity for patients. They help organise the patient's story and stimulate the patient's feeling of being understood.

Of course as a doctor you want something from your patient, but you also have to give something back. Some sort of assurance that you really understand what that person wants from you.

Student/VU

##### Less focus on the 'here and now'

There is less focus on the 'here and now', because so much attention goes to structure, and the structure is not flexible. It is tempting to move from one recommendation to the next, without paying attention to clues provided by the patient.

#### Other impact: positive effects

##### Higher quality of consultations

The users think that guideline use improves doctor patient communication in many cases. Exploring the request for help in particular is considered to result in better-helped patients. Exploring emotions and giving feedback on them to patients is believed to reduce somatisation.

Whenever I feel that the interview did not go very well, that's usually the problem. Then I think well it was not clear after all why she really came to see me. You have talked with someone for ten minutes and you didn't discuss what it really was all about.

GP trainee/Nijmegen

##### The guideline does justice to both patient and doctor

Both patients' wishes and doctors' needs are being attended to. Responsibility for the consultation is shared and deliberation about the course of action is stimulated.

What I like is the dynamic of it, there's a sort of dynamic in first actively listening and then taking action and using your expertise and then looking at the situation together like well is this what's going on. And I think that's, well I'm very comfortable doing things this way that you're actually sharing, the responsibility for the consultation and to me that's the main thing.

GP trainer/VU

##### Less chance of doctors jumping to conclusions

The guideline helps to prevent doctors from *jumping to conclusions*, because it forces them to explicitly check hypotheses about the disease or the request for help.

##### Fewer unreasonable patients

Once they have started to use the guideline, doctors find that they come to see fewer patients as unreasonable.

Since I started using the consultation model, I haven't encountered that sort of problem and I see hardly any unreasonable patients. And when a patient is unreasonable I know exactly what the matter is.

GP trainee/UVA

#### Other impact – negative effects

##### Loss of time

This barrier is specifically mentioned when participants explain why they do not adhere to communication guidelines, mostly in combination with the statement that different situations need a different approach. Doctors feel that the guidelines pay little heed to efficiency. Due to the absence of clearly stated priorities, all of the recommendations seem equally important in every situation. Doctors tend to think, however, that the relevance of the recommendations varies among consultations. Using all of the recommendations takes more time than doctors feel they have available for a consultation. This barrier is counterbalanced by the opinion that using the guideline structure saves time and promotes time management, especially in complex consultations. The users also say that it saves time to work on the patient's request for help. However, all things considered, most doctors feel that using the guideline takes more time than it saves.

When you have ten minutes per patient and every time you have to ask about course, reaction, reaction to the diagnosis. Well, what do you think? I will give you some eardrops, is that alright with you? Then I think, that's going to take far too much time.

GP trainee/Nijmegen

##### Loss of natural interaction and personal style

This is due to the strong focus on structure and having to ask very focused questions and repeat them to check whether the patient has understood what was said. This leaves little room for experiment.

I'm not a very structured person myself. When I see that I think well that's not my way of treating people. There's no fun left in my consultations this way.

GP trainee/UVA

All normal interaction disappears. For that's not something you would normally do, when you say A I think you mean A and I'm not going to ask you well do you really mean A? It's as if you're running off a list.

GP trainee/Nijmegen

##### Creating anxiety

The guideline stimulates doctors to explore patients' emotions, but in some cases exploration of anxiety causes unrest in patients.

Sometimes people say, should I be afraid or is there something I should be worried about? Then I think, oh dear, now I've really frightened them. So sometimes patients just don't get it at all.

GP trainee/Nijmegen

### Suggestions for improvement

#### Suggestions for additions or deletions

For one of the guidelines a addition and a deletion were suggested. It was suggested to add reflection on previous consultations about the same problem in order to emphasise continuity of care. The suggested deletion involved shortening the 'agenda setting phase' to merely listing the items on the agenda of the consultation, because negotiating over the consultation agenda was considered to be necessary in very rare cases only.

#### Suggestions for improvement of the development procedure

The smallest recommended change was to update the supporting literature. A more drastic suggestion was to develop guidelines that are evidence based instead of consensus based. The evidence should preferably be derived from general practice. There should be evidence of the effectiveness of communication guidelines with regard to patient outcomes, cost effectiveness and patient and doctor satisfaction.

I would be very happy when models were designed that are really derived from theory, based on empirical studies of consultations in general practice, so not laboratory conditions, but based on GPs' practice routine. What has been developed so far, was generally developed in the laboratory.

GP trainer/VU

#### Suggestions to develop different types of guideline

One suggestion was to develop a guideline for long-term patient management strategies. All the other suggestions relate to the development of guidelines that offer more flexibility in the use of communication strategies. It was also suggested to develop different guidelines for different types of patients, a guideline not intended for integral use, a guideline with different courses of action depending on the situation and a guideline showing the consequences of non-adherence, which was thought to help doctors in making considered decisions.

This is a model of a first consultation, you want to have models for different consultations, or types of consultation. Or a model especially suitable for a long-term doctor patient relationship, a chronic disease or something like that.

GP begeleider/VU

I would rather like a model that would reveal why ... for instance when you forget to explicitly pursue a certain line of questioning ... that it would reveal whether that is really bad for the patient's follow-up.

Student/VU

## Discussion

In this focus group study, we interviewed users of communication guidelines in order to explore facilitating and impeding characteristics of guidelines in respect of the actual utilisation of these guidelines. The aim was to identify ways of making guidelines more user centred and thereby promote guideline adherence.

Our participants spent more time discussing impeding than facilitating guideline characteristics. The most frequently mentioned impeding characteristics are the assumptions underlying guideline development, which are thought not to correspond with routine day-to-day practice. As a result doctors feel that there are many situations in which the guideline is difficult to apply. The rigidity of guidelines is specifically mentioned as a reason for non-adherence to communication guidelines, because it runs counter to the individualised communication approach that GPs commonly use. The users indicated that this rigidity makes guideline usage inefficient and time consuming. Less frequently mentioned impeding characteristics are flaws in the development procedure, such as lack of proof of effectiveness in general practice. The facilitating characteristic that was mentioned most frequently is the phased structure, which helps GPs manage the consultation. Positive effects of using the guideline such as better consultations or doing justice to both doctor and patient were also mentioned, albeit less often.

Most suggestions for improvement concern ways to change the main impeding characteristics: developing flexible guidelines that are applicable in many situations and that have proven effectiveness for the settings in which they are to be used. The similarity of the results for the different guidelines suggests that, despite differences, all the guidelines have the same strengths and weaknesses, i.e. supporting structure but limited applicability.

We will look at these results in light of the literature. The most common complaint uttered by the focus group participants concerned incorrect assumptions underpinning guideline development and flaws in the development process. An instrument to assess the quality of the dutch communication guidelines indicated poor guideline quality, especially as regards development rigour, user involvement and applicability[16] This suggests that guideline users are probably right in complaining about lack of evidence base. Lack of user involvement and attention for applicability in guideline development increase the likelihood of erroneous assumptions underpinning guidelines, with detrimental effects on the applicability of guidelines.

We will now take a closer look at the two most frequently mentioned complaints regarding inaccurate assumptions. The first complaint is that guidelines that advocate shared decision making are not equally appropriate for all patients, because not all patients can be equal and willing negotiation partners. There is indeed evidence that not all patients are comfortable with shared decision making [47,48]. There is also evidence that it is difficult to explain concepts regarding risks related to treatment or non-treatment of their disease to patients in such a way that they can really grasp it [49-51]. This may contribute to the participants' perceptions that some patients are not really capable of involvement in decisions. The communication guidelines discussed by the focus groups provide no recommendations as to how to act when the patient does not want to take decisions or does not seem to understand information about risks involved. This may cause doctors go to abandon patient centred guidelines in these situations. However, this may compromise patients' autonomy, because doctors have reported to have difficulty discerning which patients want to take part in decision making and which patients prefer to let the doctor take the decisions for them [52]. Generally, the results of the focus groups seem to show that doctors are rather ambivalent as regards shared decision making. Doctors prefer guidelines that take account of both the patient's and the doctor's interests. They acknowledge that it is important to explore the patient's request for help and to share decisions, but at the same time they want to remain in charge of the consultation. In assessments of doctor patient communication in daily practice, scores on items reflecting patient centredness or shared decision making tend to be low [29,53,54]. The percentages of consultations with low scores on shared decision making seem to be higher than the number of patients unwilling to take part in decision making [29,47,48]. In order to further shared decision making doctors' ambivalence towards it should be examined more extensively.

The second complaint of the focus group participants was that different situations demand different communication strategies, whereas the guidelines are generic. This view seems to be in line with the current literature. There is a wealth of communication guidelines for specific situations, such as breaking bad news, anti smoking counselling, patients with a chronic disease, and conflict situations [14,16]. The Dutch GP training centres use four guidelines for specific situations in addition to the generic ones (see appendix 1). However, neither these guidelines nor the literature offer a framework to determine which guideline to use in which situation. There are communication guidelines for different types of patients, different types of diseases and different goals of the doctor [14,16]. However, there appears to be no consensus about which characteristics of a situation determine which type of communication is most appropriate.

The facilitating factor of the guidelines that is most frequently mentioned by the participants is the structure of the consultation which is divided into conceptually different phases. Participants say that this structure gives them more grip on the consultation. This aspect of the guidelines has been little studied. However, Cegala et al. suggest that a chronological order of recommendations, following the course of the consultation, would be helpful [55]. Silverman et al. advocate organising the recommendations in a conceptual framework comprising the main tasks of a doctor in a consultation [6]. The latter proposal seems to be supported by our results, with the main tasks being similar to the 'conceptual difference' in the phases of our guidelines.

### Strengths and weaknesses

Our results are based on discussions of communication guidelines used by all but one of the Dutch medical schools in general practice settings. The fact that the results are similar for four different guidelines suggests that they may be at least partly generalisable to other communication guidelines, especially if they have a phased structure and recommend a single course of action. Doctor patient communication is argued to be essentially similar in different health care settings (for example primary or secondary care) [6], which suggests that our results should be generalisable to other health care settings. However, as our results stress the effect of the context of a consultation on the usefulness and applicability of recommendations for communication, we think it is important to explore facilitating and impeding characteristics of communication guidelines in the context of different health care settings.

In this study we made use of the expertise of different groups of guideline users, which enhanced the richness of the results. Participation was high: seven of the eight Dutch medical schools participated.

The discussions of the guidelines in the focus groups reflect users' interpretations of the guidelines. Many of the participants in the study are communication trainers. Although their interpretations may be assumed to be largely accurate, misconceptions cannot be ruled out completely.

The nature of focus group discussions implies that only those barriers and facilitating factors were discussed that the participating users were aware of. An observational study in clinical practice might reveal additional impeding or facilitating guideline characteristics.

We were interested in barriers and facilitators experienced by doctors that try to use communication guidelines. For this reason, we have not included patients in our study, in this stage. However, patients are the most important stakeholders of doctor patient communication. It is therefore important that their opinions and preferences are taken into account during development of communication guidelines.

If we want to develop communication guidelines that are user centred, applicable and evidence based, we need to resolve two problems. The first one is how to develop a set of guidelines that offers a strong and supporting structure yet at the same time allows sufficient flexibility to support communication strategies that can be tailored to individual situations. The second problem is how to develop a flexible set of guidelines to tailor communication strategies to individual consultations and support the use of evidence based communication strategies. Should we develop guidelines for different types of patients, different types of diseases, or different goals of the doctor? An answer to this question might be found in the notion that communication serves to address the goals of its participants [56,57], in our case those of doctors and patients. In order to support doctors in using effective communication strategies, we might consider developing guidelines with a conceptual structure consisting of different consultation phases, but with evidence-based recommendations within these phases that vary depending on the goal the doctor is trying to accomplish in a particular consultation. This offers three advantages: 1. The main facilitating factors of the present structure are preserved. 2. Doctors can adapt their communication strategies to the goals they think relevant for a specific consultation 3. The guidelines can incorporate evidence-based overviews of the best way to pursue specific goals. Another advantage of goal orientated communication strategies is that the effectiveness of the communication can be evaluated by checking if the goals have been achieved [56]. If these guidelines focus on goals that are specific for certain diseases, they could serve as an addition to existing medical technical guidelines. This solution is in keeping with the participants' view that goal-directed communication is an important component of medical professionalism.

## Conclusion

Although communication guidelines are considered useful, especially for structuring consultations, their feasibility is strongly impaired by lack of flexibility and applicability to practice routines. As feasible communication guidelines are an important tool for the improvement of doctor patient communication, the development of guidelines that combine a supportive structure with flexibility to tailor communication strategies to specific situations should be considered. Further research might address first how this combination of a supportive structure and flexibility can be realised best. This would allow testing whether flexible communication guidelines indeed have a higher adherence then the existing communication guidelines.

## Competing interests

P.R. is co-author of one of the guidelines [41] under discussion in this article

## Authors' contributions

WV, PR, TvdW and CvdV were involved in discussions that led to the original idea for the research. WV collected the data. WV, PR, TvdW and SN were involved in analysing the data. WV and PR wrote the first draft of the paper. All authors critically revised the paper for important intellectual content and approved the final version.

## Appendices

### Appendix 1

The guidelines for doctor patient communication, as used in the universities that participated. The guideline that was used most was topic of our discussion and is printed in bold. The titles of the guidelines have been translated into English.

Erasmus University Rotterdam

• **MAAS-global manual 2000 **^14,27^

Radboud University Medical Centre Nijmegen

• Syllabus consultations in general practice

• The five-consultations model

• **MAAS-global manual 2000 **^14,27^

• NHG-cahier 1: How may I help you? Why patients visit their GP.

Leiden University

• **MAAS-global manual 2000 **^14,27^

Maastricht University

• **MAAS-global manual 2000 **^14,27^

• A model for the structure of a consultation

• Calgary-Cambridge guides ^25^

• 6 step conflict management model

University of Amsterdam

• **The consultation model**

• Phasing of a bad news conversation

• Talking about errors

• Counselling conversation model

Utrecht University

• No title, 176 pages, Syllabus year 1

• **Laconto**

• A bad news model

• Managing medically unexplained complaints

Free university of Amsterdam

• **Syllabus: consultations in general practice**

• MAAS-global manual 2000 ^14,27^

• Model for the bad news conversation

### Appendix 2

List of guidelines discussed in this study, with short descriptions of the guideline

1. Laconto. J.C.M. Bloemen, L.H.C. Tan. Utrecht: SVUH. 1994

*Theoretical background: *not described, content appears to be patient centred

*Content: *Combines recommendations for a focused and systematic consultation with those for doctor patient communication.

*Structure: *There are three chronological consultation phases, with six to seven recommendations for good GP-patient communication within each phase.

*Situation: *the guideline is meant for all consultations in general practice.

2. MAAS-global manual (Dutch version). Jacques van Thiel, Paul Ram, Jan van Dalen Maastricht: Maastricht University. 2000. 

*Theoretical background: *not described, content appears to be patient centred. Good doctor patient communication is defined as the situation in which both parties are seeking to align their mutual goals and are aware of the meaning of the information exchanged.

*Content: *Combines recommendations for doctor patient communication with recommendations for medical technical skills.

*Structure: *There are recommendations for separate consultation phases (seven phases), general communication (six items) and medical technical skills (4 items).

*Situation: *The guideline is meant for consultations that are relatively complete and uncomplicated, such as when the patient presents with only one complaint and the consultation comprises all phases.

3. Syllabus: consultations in general practice. Marion Schmitz, Chris Claus. Amsterdam: Free University of Amsterdam. 2000.

*Theoretical background: *not described, content appears to be patient centred.

*Content: *Recommendations for doctor patient communication.

*Structure: *There are recommendations for four separate consultation phases. The goals that should preferably be achieved by following these recommendations are described for each phase.

*Situation: *Not described, the guideline seams to be meant for GP-patient consultations in general.

4. The consultation model. Amsterdam: University of Amsterdam. Author and date not mentioned.

*Theoretical background: *not described, content appears to be patient centred.

*Content: *Recommendations for doctor patient communication and recommendations regarding medical problem solving.

*Structure: *There are recommendations for three separate consultation phases. For each phase the appropriate attitude towards the patient is described.

*Situation: *Not described, the guideline seams to be meant for GP-patient consultations in general.

### Appendix 3

Interview scheme

The chairman made sure the following topics were discussed, with regard to the use of the guideline in clinical practice and with regard to the guideline as a teaching instrument. In this paper the results that consider the use of the guideline in clinical practice have been described only.

1. The manner in which the guideline is used (this topic was added after it had been discussed spontaneously in the first three interviews)

2. The strengths of the guideline

3. The weaknesses of the guideline

4. Ways to improve guidelines for DPC

To stimulate discussion the chairman could offer the following questions:

• How does the guideline help you?

• In which situations does the guideline not provide enough help? What help could you use in that situation?

• Are their situations in which it is hard to apply the guideline? Why is that difficult?

• How can this guideline be improved?

• What would a better guideline look like?

## Pre-publication history

The pre-publication history for this paper can be accessed here:


